# Developmental venous anomaly associated with dural arteriovenous fistula: Etiopathogenesis and hemorrhagic risk

**DOI:** 10.3389/fsurg.2023.1141857

**Published:** 2023-03-21

**Authors:** Edoardo Agosti, Lucio De Maria, Pier Paolo Panciani, Simona Serioli, Dikran Mardighian, Marco Maria Fontanella, Giuseppe Lanzino

**Affiliations:** ^1^ Division of Neurosurgery, Department of Medical and Surgical Specialties, Radiological Sciences and Public Health, University of Brescia, Brescia, Italy; ^2^ Division of Neuroradiology, Department of Medical and Surgical Specialties, Radiological Sciences and Public Health, University of Brescia, Brescia, Italy; ^3^ Department of Neurologic Surgery, Mayo Clinic, Rochester, MN, United States

**Keywords:** intracranial vascular malformation, developmental venous anomaly, dural arteriovenous fistula, etiopathogenesis, hemorrhagic risk

## Abstract

**Introduction:**

Developmental venous anomalies (DVAs) have traditionally been defined as non-pathological congenital lesions. Compared to isolated DVAs, the association of DVAs with arteriovenous shunts seems to have a more adverse clinical connotation. In this review, we describe the association between DVA and dAVF and discuss the hemorrhagic risk. We also advance a hypothesis about the potential *de novo* formation of a DVA and challenge the dogma about their “developmental” or “congenital” nature.

**Methods:**

A systematic review of the literature on the association of DVA and dAVF was performed in accordance with the PRISMA-P (Preferred Reporting Items for Systematic Review and Meta-Analysis Protocols) guidelines.

**Results:**

A number of 678 papers was initially identified, but only 9 studies were included in the final qualitative analysis. Most of the patients presented with bleeding (56%), with a median GCS of 14 (range 10–15). In 56% of the cases the DVA had a supratentorial location. Supratentorial DVAs mostly drained in the superior sagittal sinus (80%), while all of infratentorial/combined DVAs drained in deep ependymal veins of the 4th ventricle. All the supratentorial dAVFs drained into the superior sagittal sinus, while the infratentorial/combined dAVFs mostly drained in the jugular bulb, Vein of Rosenthal, or transverse-sigmoid sinuses (75%). Most of the dAVFs were classified as Cognard type IIa + b (67%), while in a smaller number of cases type I (22%) and type V (11%). The dAVF was the target of treatment in each case and most patients underwent endovascular treatment (78%). The dAVF was completely occluded in 78% of cases and no periprocedural complications were reported.

**Conclusion:**

The clinical presentation, radiological findings, and treatment outcomes of DVAs and associated dAVFs have been discussed. Despite the general opinion that DVAs are benign congenital lesions, increasing epidemiological and radiological evidence supports a potential acquired origin, and the venous system seem to play a pivotal role in their post-natal genesis and development.

## 1. Introduction

Developmental venous anomalies (DVAs) have traditionally been defined as non-pathological congenital lesions (1). Various hypotheses on DVAs etiology have been proposed but there is no consensus as to their genesis and clinical significance. Historically, the cerebral venous system (CVS) has been considered a “passive” component of vascular malformations. However, recent evidence suggests a potential role of the CVS in the genesis of intracranial vascular malformations (IVMs) (2–5).

Classically, the intracranial vascular malformations (IVMs) have been classified by McCormick into four distinct groups, including arteriovenous malformation (AVM), cavernous malformations (CM), DVA (“venous angiomas”), and capillary telangiectasia (1). Nonetheless, the observation over time that IVMs can occur in association or hybrid forms has raised questions about their potential common genesis (6). The association between DVA and dAVF has been occasionally reported over the years (7–15). Compared to isolated DVAs, the association of DVAs with arteriovenous shunts seems to have a more adverse clinical connotation than the classical isolated DVAs (16).

In this review, we describe the association between DVA and dAVF, advance a hypothesis about the potential *de novo* formation of a DVA, and challenge the dogma about their “developmental” or “congenital” nature.

## 2. Methods

This study was conducted in accordance with the PRISMA-P (Preferred Reporting Items for Systematic Review and Meta-Analysis Protocols) guidelines (17). A systematic review of the literature on the association of DVA and dAVF was performed. An online literature search was launched on PubMed, Medline, and Scopus using the following research string: “[(dural arteriovenous fistula or dAVF) AND (developmental venous anomaly OR DVA OR cerebral venous malformations OR venous angiomas)]”. The last search for articles pertinent to the topic was conducted on December 31st, 2022. Other pertinent articles were retrieved through reference analysis. Two authors (EA and LDM) independently conducted the abstract screening for eligibility. Any discordance was solved by consensus with two senior authors (MMF and GL). No restrictions on the date of publication were made. Exclusion criteria were as follows: studies published in languages other than English, studies including hybrid forms of dAVF and DVA (i.e., arterialized DVAs, DVAs with arteriovenous shunts, atypical AVMs with DVA, transitional forms between DVAs and AVMs, AVMs draining into DVA, and AVMs with venous predominance), studies missing methods details, studies without detailed neuroimaging and a clear description of the vascular anatomy of the IVMs. Inclusion criteria: studies reporting at least a case of DVA associated with dAVF.

## 3. Results

### 3.1. Literature search

**Figure 1:**
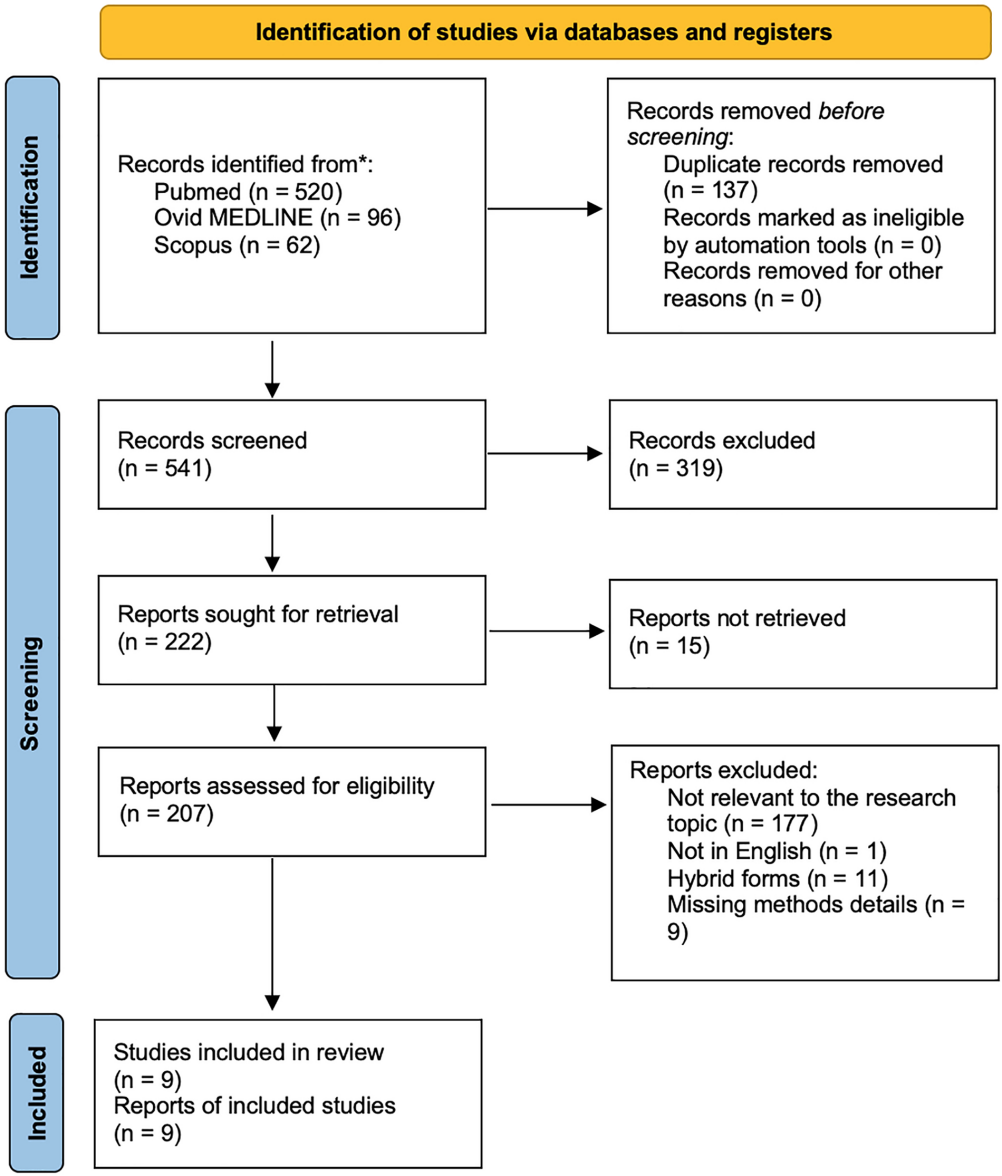
PRISMA diagram showing the research strategy and selection of papers included.

We identified 678 papers using the reported keywords. After removing 137 duplicates, we examined all 541 abstracts and obtained 207 full-text eligible articles. We excluded additional 198 studies, which did not meet the inclusion criteria. The final qualitative analysis was conducted on 9 studies. Figure 1 shows the paper selection according to PRISMA.

### 3.2. Literature search

A total of 9 patients, 6 males (68%) and 2 females (22%), were included in the final quantitative analysis. The median age at presentation was 18 (range 3–32). Most of the patients presented with bleeding (5; 56%), 3 presented with symptoms unrelated to the rupture of the vascular malformation (33%), and one was discovered incidentally (11%). Specific presentation of each case is reported in Table 1. The median GCS at presentation was 14 (range 10–15). In 5 cases the DVA had a supratentorial location (56%), while in 4 infratentorial or combined location (44%). Supratentorial DVAs drained in the superior sagittal sinus (4; 80%) and transverse sinus (1; 20%), while all of infratentorial/combined DVAs drained in deep ependymal veins of the 4th ventricle. The annexed dAVFs drained into the superior sagittal sinus when supratentorial (5; 100%), and in the jugular bulb, Vein of Rosenthal, or transverse-sigmoid sinuses when infratentorial/combined (3; 75%). Noteworthy, one infratentorial dAVF (1; 25%) drained into the DVA. Two dAVFs were classified as Cognard type I (22%), 6 type IIa + b (67%), and one as type V (11%). The dAVF was the target of treatment in each case. Seven patients underwent endovascular treatment (78%) and two surgical treatment (22%). The dAVF was completely occluded in 7 cases (78%) and no periprocedural complications were reported. The median mRS after the procedure was 0 (range 0–2) and only 3 patients continued to have residual symptoms at follow-up (33%). The median length of follow-up was 36 months (range 4–72 months). Table 1 summarizes the results.

**Table 1:** Summary of the results.

Author, journal, year	Age, gender	Initial presentation			Location and vascular anatomy					Treatment		Post-treatment presentation		Follow up (months)
					dAVF				DVA					
		General	GCS	Symptoms	Location	Feeders	Draining veins	Cognard type	Drainage	Procedure	Result	mRS	Residual Symptoms	
**Hirata, Surg Neurol, 1986**	18, M	Hemorrhage	14	Left hemiparesis, Left sensory disturbance	Right parietal lobe	Central parietal artery, Anterior parietal artery	Superior sagittal sinus	IIa + b	Superior sagittal sinus	Surgery	No residual dAVF	2	Left hemiparesis	24
**Kuncz, Acta Neurochir (Wien), 2001**	31, M	Unruptured Symptomatic	10	Grand mal seizure, Drowsiness, Expressive dysphasia, Right hemiparesis	Bilateral frontal lobes	Ethmoidal arteries	Superior sagittal sinus	IIa + b	Superior sagittal sinus	Surgery	No residual dAVF	2	Right hemiparesis	72
**Dudeck, J Neurosurg, 2004**	16, M	Hemorrhage	13	Dysarthria Ataxia, Blurred vision, Left hemiparesis	Right cerebellar hemisphere	Right cerebellar arteries, Right occipital artery, Right ascending pharyngeal artery	Right transverse-sigmoid sinuses	IIa + b	Deep ependymal vein of the 4th ventricle	Embolization (Onyx)	Residual dAVF	0	None	36
**Fok, Interv Neuroradiol, 2006**	6, M	Hemorrhage	13	Severe occipital headache, Vomiting, Right dysmetria	Right cerebellar hemisphere	Superior cerebellar artery	DVA	V	Deep ependymal vein of the 4th ventricle	Embolization (Onyx)	No residual dAVF	1	None	4
**Geibrasert, Interv Neuroradiol, 2007**	3, F	Unruptured Asymptomatic	15	Incidental finding after trauma	Right frontoparietal lobe	Right middle meningeal artery, Right superior temporal artery	Superior sagittal sinus	IIa + b	Superior sagittal sinus	Embolization (NBCA)	No residual dAVF	0	None	72
**Roh, Korean J Radiol, 2012**	18, M	Hemorrhage	13	Drowsiness, Vomiting, Left hemiparesis	Right parietal lobe	Right pericallosal artery	Superior sagittal sinus	IIa + b	Superior sagittal sinus	Embolization (NBCA)	No residual dAVF	2	Left hemiparesis	60
**Chakravarthy, Neuroradiol J, 2016**	27, M	Unruptured Symptomatic	15	Left eye proptosis	Left sphenoid region, Left cerebellar hemisphere, Brainstem	Left middle meningeal artery, Left internal maxillary artery, Meningeal branches of the left cavernous ICA, Left ophthalmic artery	Vein of Rosenthal, Cavernous sinus, Inferior petrosal sinus	I	Deep ependymal vein of the 4th ventricle	Embolization (Onyx)	No residual dAVF	0	None	NR
**Brinjikji, World Neurosurg, 2020**	26, M	Unruptured Symptomatic	15	Incidental finding due to left parietal scalp lesion	Right basal ganglia, Capsular region, Torcular Herophili	Posterior meningeal artery, Meningohypophyseal trunk	Deep venous system, Superior sagittal sinus	I	Bilateral transverse sinuses	Embolization (Onyx)	No residual dAVF	0	None	NR
**Hashikata, J Stroke Cerebrovasc Dis, 2022**	32, F	Hemorrhage	15	Ataxia	Bilateral cerebellar hemispheres	Left occipital artery	Left jugular bulb	IIa + b	Deep ependymal vein of the 4th ventricle	Embolization (coils)	No residual dAVF,, Residual DVA	0	None	36

## 4. Discussion

### 4.1. Discussion of main findings

In this literature review we found 9 cases of DVA associated with a dAVF. In most cases (56%) they presented with hemorrhage and were allocated in the supratentorial compartment. Treatment was more commonly through endovascular approach which was effective with complete occlusion of the dAVF in most cases without complications.

DVAs have been classically described by McCormick et al. (1) as a composition of radial veins with interspersed neural parenchyma converging toward a central enlarged collector, draining normal cerebral tissue. Since the DVA participates in the drainage of normal parenchyma, surgical compromise or occlusion of the DVA often results in massive venous infarcts in the corresponding drained territory (10, 16). Although DVAs do not require treatment *per se*, it has been suggested that when associated with arteriovenous shunts or AVMs, the presence of an associated DVA confers higher hemorrhagic risk and requires more aggressive treatment. Accordingly, we found a high prevalence (56%) of hemorrhagic presentation in the case of DVA associated with dAVFs. This can be related to increased venous pressure of arterialized DVA secondary to fistulization that can predispose to its rupture (12).

The most common vascular anomaly associated with DVA is CM (from 8 to 33% of patients with DVA) (3), and DVAs are thought to play a causative role in the formation of sporadic CMs (18). Less common is the association of DVA with capillary telangiectasia or AVM (2, 19). The association of DVA and dAVF seems to be the least common and, as revealed by our literature review, only 9 cases have been reported over the years (7–15). On the other hand, several reports describing hybrid IVMs consisting of atypical DVA with arteriovenous shunts have been reported. These mixed IVMs have been variously described as arterialized DVAs, DVAs with arteriovenous shunts, atypical AVMs with DVA, transitional forms between DVAs and AVMs, AVMs draining into DVA, and AVMs with venous predominance (12, 20–22). For instance, Mullan et al. (21) presented a particularly detailed analysis of the angiographic characteristics of DVAs associated with arteriovenous shunts. Im et al. (22) reported a series of 15 patients with atypical DVAs with arteriovenous shunts.

**Figure 2:**
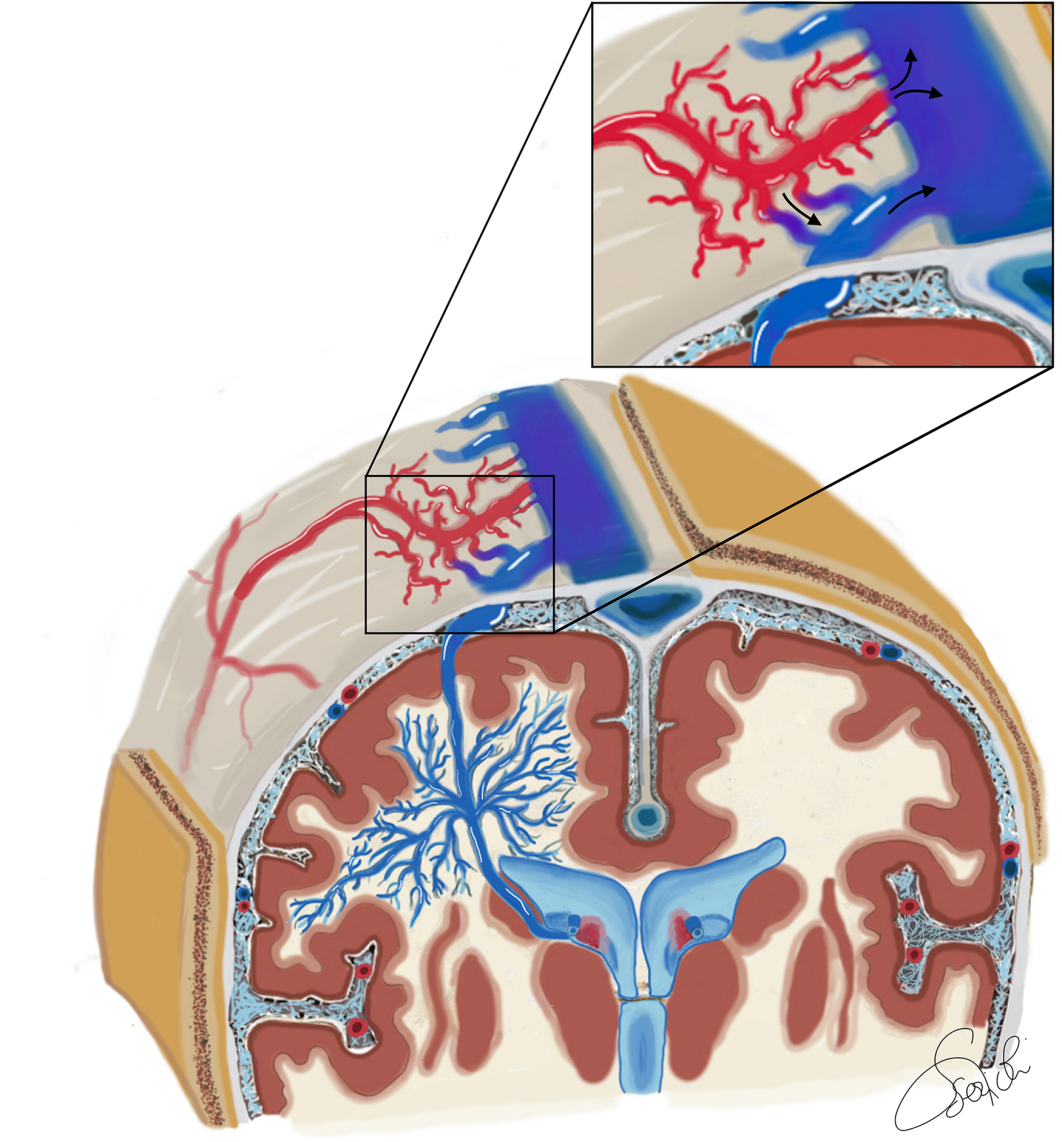
Schematic illustration of the vascular angioarchitecture of a superficially draining DVA associated with a type 2 a + b dAVF in a coronal section through the region of the foramen of Monro, with anteroposterior view of the bilateral cerebral hemispheres. The deep white matter principally drains in an anomalous centrifugal fashion toward the superficial cerebral venous system. These veins converge into a single vein called the collecting vein of the DVA, and form the so-called caput medusae. The collecting vein serves as the major venous outflow of the DVA and continues as a dilated cortical vein that joins the superior sagittal sinus. A connection between the DVA and the subependymal veins has also been depicted. The DVA collecting cortical vein has a fistula connection with the arterial portion of the dAVF. Hence the dAVF is a type II a + b according to Cognard, having both a direct drainage into the superior sagittal sinus and a discharge into the cortical venous system of the DVA.

Although it is commonly thought that DVAs are congenital vascular lesions, the actual etiology remains uncertain. Many hypotheses have been postulated, including abnormalities in venous development or physiological response to thrombosis of superficial or deep collecting veins during embryogenesis or early life. However, recent epidemiological and radiological data proposed a possible acquired origin (10, 16). Brinjikji et al. (3) showed that DVAs prevalence increases significantly in the first ten years of life (1.5% to 9.6%), postulating that DVAs may form in the post-natal period as a functional adaptation of the CVS to local venous thrombosis (3, 14). In accordance with the “double hits theory”, venous pathways would be the main promoter of DVA genesis, adapting their angioarchitecture in response to local thrombotic events as trigger of proangiogenic susceptibility (2). Chakravarthy et al. (13) reported a case of a high-flow sphenoid wing dAVF associated with the later development of an adjacent DVA in the temporal lobe. Similarly, Brinjikji et al. (14) described a case of torcular dAVF associated with the subsequent development of multiple adjacent DVAs increasing in size over time as the dAVF developed more aggressive angioarchitecture features. Both of these radiological reports seem to document how the increased venous hypertension in the superficial venous system from the dAVF likely resulted in the growth and evolution of the DVA. Therefore, the DVA would represent a dynamic compensatory response to locoregional venous flow alterations induced by the dAVF (Figure 2). Their pivotal role as an acquired compensatory connection between the deep and the superficial CVS is demonstrated both by the absence of normal venous drainage around the DVA and by the disastrous consequences related to their surgical closure (14).

Other authors reported dAVFs with coexisting or preexisting DVA. Of note, a reverse mechanism of dAVF induced by DVA can be postulated. Accordingly, spontaneous thromboses of a DVA draining veins have been reported in the literature and several case reports documented arteriovenous shunt formation after DVA thrombosis. Consequently, it is reasonable to suspect that thrombosis of the whole or part of the medullary or collector veins might induce fistulization. Wilson et al. speculated that inconspicuous thrombosis of one of the radial veins of the DVA could increase intravenous pressure within a DVA and promote dAVF *de novo* formation. Agazzi et al. described a case of venous thrombosis in a system of two distinct DVAs, one of which subsequently formed a dural arteriovenous shunt. Similarly, Dudeck et al. (9) and Mullan et al. (21) both described a DVA associated with the subsequent appearance of a dAVF. It remains unclear whether this association between the DVA and *de novo* formation of the adjacent dAVF represents a true cause-and-effect relationship or a coincidental expression of an inappropriately formed cerebral vasculature (21).

### 4.2. Limitations

The small number of cases reported in the literature is among the limitations of this study. The variable quality of reported cases, especially regarding vascular angioarchitecture of IVMs, might have influenced the results and sometimes complicated classification of malformations in DVA associated with dAVF, arterialized DVA, or AVMs draining into DVA (6). Nonetheless, our study provides helpful information for providers considering the treatment of DVAs with associated dAVFs, and provides guidance for future areas of investigation.

## 5. Conclusion

We reviewed the literature about the little-known association between DVAs and dAVFs. The clinical presentation, radiological findings, and treatment outcomes of DVAs and associated dAVFs have been discussed. The authors also queried the potential role of the alteration of venous outflow in the genesis of either lesion. Further analysis of similar cases may better clarify the significance and role of the DVAs. Despite the general opinion that DVAs are benign congenital lesions, increasing epidemiological and radiological evidence supports a potential acquired origin, and the venous system (i.e., deep and superficial cerebral venous compartments) seem to play a pivotal role in their post-natal genesis and development.

## Data Availability

The original contributions presented in the study are included in the article/Supplementary Material, further inquiries can be directed to the corresponding author.
